# Lentiviral Hematopoietic Stem Cell Gene Therapy Rescues Clinical Phenotypes in a Murine Model of Pompe Disease

**DOI:** 10.1016/j.omtm.2020.07.001

**Published:** 2020-07-06

**Authors:** Giuseppa Piras, Claudia Montiel-Equihua, Yee-Ka Agnes Chan, Slawomir Wantuch, Daniel Stuckey, Derek Burke, Helen Prunty, Rahul Phadke, Darren Chambers, Armando Partida-Gaytan, Diego Leon-Rico, Neelam Panchal, Kathryn Whitmore, Miguel Calero, Sara Benedetti, Giorgia Santilli, Adrian J. Thrasher, H. Bobby Gaspar

**Affiliations:** 1 Infection, Immunity and Inflammation Program, Molecular and Cellular Immunology Section, UCL Great Ormond Street Institute of Child Health, University College London, London WC1N 1EH, UK; 2 Centre for Advanced Biomedical Imaging, University College London, London WC1E 6DD, UK; 3 Enzyme and Metabolic laboratory, Great Ormond Street Hospital, London WC1N 3JH, UK; 4 Dubowitz Neuromuscular Centre, MRC Centre for Neuromuscular Diseases, UCL Great Ormond Street Institute of Child Health, London WC1N 1EH, UK; 5 NIHR Great Ormond Street Hospital Biomedical Research Centre, London WC1N 1EH, UK; 6 Orchard Therapeutics Ltd., London EC4N 6EU, UK

**Keywords:** Pompe disease, hematopoietic stem cell gene therapy, lentiviral vector, acid alpha-glucosidase, GAA, erythroid-specific enhancer, lysosomal storage disorders, personalized medicine

## Abstract

Pompe disease is a lysosomal storage disorder caused by malfunctions of the acid alpha-glucosidase (GAA) enzyme with a consequent toxic accumulation of glycogen in cells. Muscle wasting and hypertrophic cardiomyopathy are the most common clinical signs that can lead to cardiac and respiratory failure within the first year of age in the more severe infantile forms. Currently available treatments have significant limitations and are not curative, highlighting a need for the development of alternative therapies. In this study, we investigated the use of a clinically relevant lentiviral vector to deliver systemically GAA through genetic modification of hematopoietic stem and progenitor cells (HSPCs). The overexpression of GAA in human HSPCs did not exert any toxic effect on this cell population, which conserved its stem cell capacity in xenograft experiments. In a murine model of Pompe disease treated at young age, we observed phenotypic correction of heart and muscle function with a significant reduction of glycogen accumulation in tissues after 6 months of treatment. These findings suggest that lentiviral-mediated HSPC gene therapy can be a safe alternative therapy for Pompe disease.

## Graphical Abstract

**Figure:**
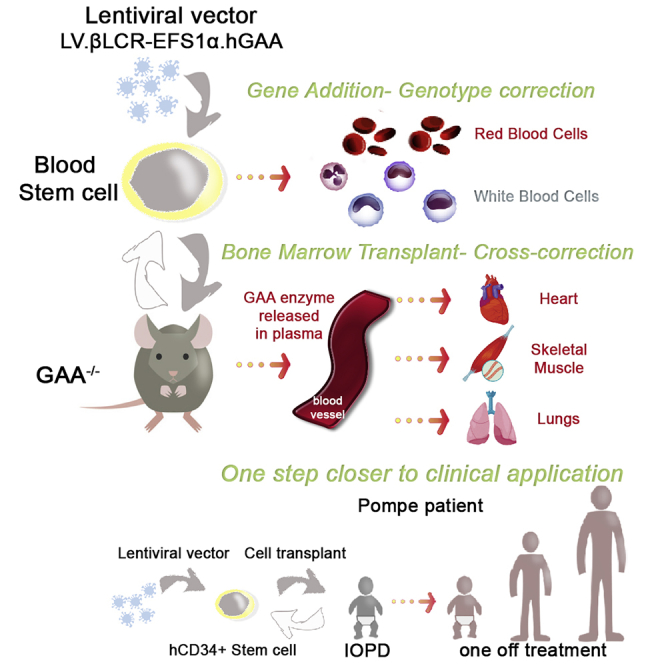

## Teaser

Pompe disease (PD) is a multisystem pathology that lacks a corrective therapy. In this study, we show that hematopoietic stem cell gene therapy by using a clinically suitable lentiviral vector designed for PD is a safe and promising therapeutic strategy that exploits genetically corrected blood cells to rescue the pathology throughout the body.

## Introduction

Pompe disease (PD; OMIM: 232300) is a rare inherited metabolic disorder resulting from an impaired metabolism of glycogen in cells. In a healthy individual, the lysosomal acid alpha-glucosidase (GAA; P10253) catabolizes α-1,4- and α-1,6-glycosidic bonds of glycogen to release its simpler component, glucose.^1^^,^^2^ In Pompe disease, mutations in the *GAA* gene leading to the absence or reduction of GAA activity results in abnormal accumulation of glycogen in lysosomes, which causes cell and tissue vacuolization and progressively leads to tissue disruption.^3^ Pompe disease is a multisystem disorder, with skeletal and cardiac muscles being the most prominently affected tissues, but other systems, including the central nervous system (CNS), can also be affected.^1^^,^^3^, ^4^, ^5^, ^6^, ^7^ According to severity, age of onset, and disease progression rate, Pompe disease has been categorized into the classic infantile onset (IOPD) and the late onset (LOPD). IOPD starts early in life and primarily manifests as hypotonia, hypertrophic cardiomyopathy, and generalized muscle weakness, leading to death in the first year of life if left untreated.^4^^,^^6^^,^^8^ In a milder spectrum, LOPD can present at any age as a progressive skeletal and respiratory muscle dysfunction leading to motor impairment and respiratory failure.^4^^,^^6^^,^^8^^,^^9^

The current and only treatment option is enzyme replacement therapy (ERT) with recombinant human GAA (rhGAA), but this is not curative and is unable to treat the nervous system due to the relative inefficiency of ERT to cross the blood-brain barrier.^6^^,^^7^^,^^10^, ^11^, ^12^, ^13^ ERT can be associated with high immunogenicity, especially in patients with the most severe form and no sign of residual enzymatic activity.^14^ Alternative treatment options that provide higher levels of muscle correction and can treat CNS abnormalities may offer significant benefits for affected individuals. Several studies have used adeno-associated viruses (AAVs) as a gene therapy delivery system to target via local or systemic administration specific tissues such as skeletal muscles, liver, and CNS.^7^^,^^15^^,^^16^ AVV-mediated gene therapy can trigger antibody or cell-mediated immune responses to transgene or viral capsid,^16^, ^17^, ^18^, ^19^, ^20^ as has been observed in clinical trials for hemophilia B^21^ using AAV, but liver-directed transgene expression has been shown in pre-clinical studies^22^ to potentially overcome this safety concern by induction of immune tolerance. Although AVV-mediated gene therapy remains a promising treatment, the episomal nature of viral persistence results in a dilution effect in replicating cells such as hepatocytes, particularly in younger patients, and thereby increasing safety concerns would arise for multiple dosages in those patients.^18^^,^^22^^,^^23^

Autologous hematopoietic stem and progenitor cell (HSPC) gene therapy has shown successful correction of the underlying phenotypes associated with primary immunodeficiency diseases, specific metabolic disorders, and hemoglobinopathies, resulting in approved therapies.^24^, ^25^, ^26^ The use of lentiviral vector (LV)-mediated HSPC gene therapy in X-linked adrenoleukodystrophies (ALDs) or metachromatic leukodystrophies (MLDs) patients demonstrated that the correction of hematopoietic cells and overexpression of the therapeutic enzyme was sufficient to stop the progressive neurodegeneration due to demyelination typically observed in the clinical course of these diseases.^27^, ^28^, ^29^, ^30^, ^31^ Similarly, promising results have been shown in pre-clinical studies in other lysosomal storage disorders such as globoid cell leukodystrophy^32^ or mucopolysaccharidosis (MPS) diseases (MPS-I, MPS-II, or MPS-IIIA/B).^33^, ^34^, ^35^, ^36^, ^37^, ^38^, ^39^, ^40^ In these cases, the overexpression of the therapeutic gene in the HSPCs reduced the toxic accumulation of metabolites in affected tissues, ameliorating or even reverting, in the case of MPS-I, disease manifestations. Two previous studies looked at the feasibility of HSPC gene therapy in Pompe disease and showed a relevant decrease of glycogen accumulation in affected tissues upon treatment as well as the induction of immune tolerance versus hGAA in treated mice.^41^^,^^42^ Although they showed promising results, the level of correction in muscles and the level of glycogen reduction shown were incomplete and demonstrated the need for further improvements to achieve greater efficacy. Furthermore, the vector or the experimental design in these two studies was not suitable for clinical translation.

To address these issues, we designed and tested a clinically relevant lentiviral construct that expresses hGAA in HSPCs and their progeny using a mammalian constitutive promoter and a previously studied regulatory cassette that overexpresses the transgene specifically in the erythroid lineage.^43^ In this study, we showed therapeutic efficacy of this clinically relevant LV in the context of HSPC gene therapy in a murine model of Pompe disease. In addition, we showed that the hGAA overexpression in human (h)CD34^+^ cells did not affect their stemness, engraftment, and differentiation *in vivo* as well as *in vitro*. Altogether, these data encourage the use of lentiviral HSPC gene therapy for the treatment of Pompe disease, in particular for the IOPD form.

## Results

### Clinically Relevant LV for Pompe Disease

**Figure 1:**
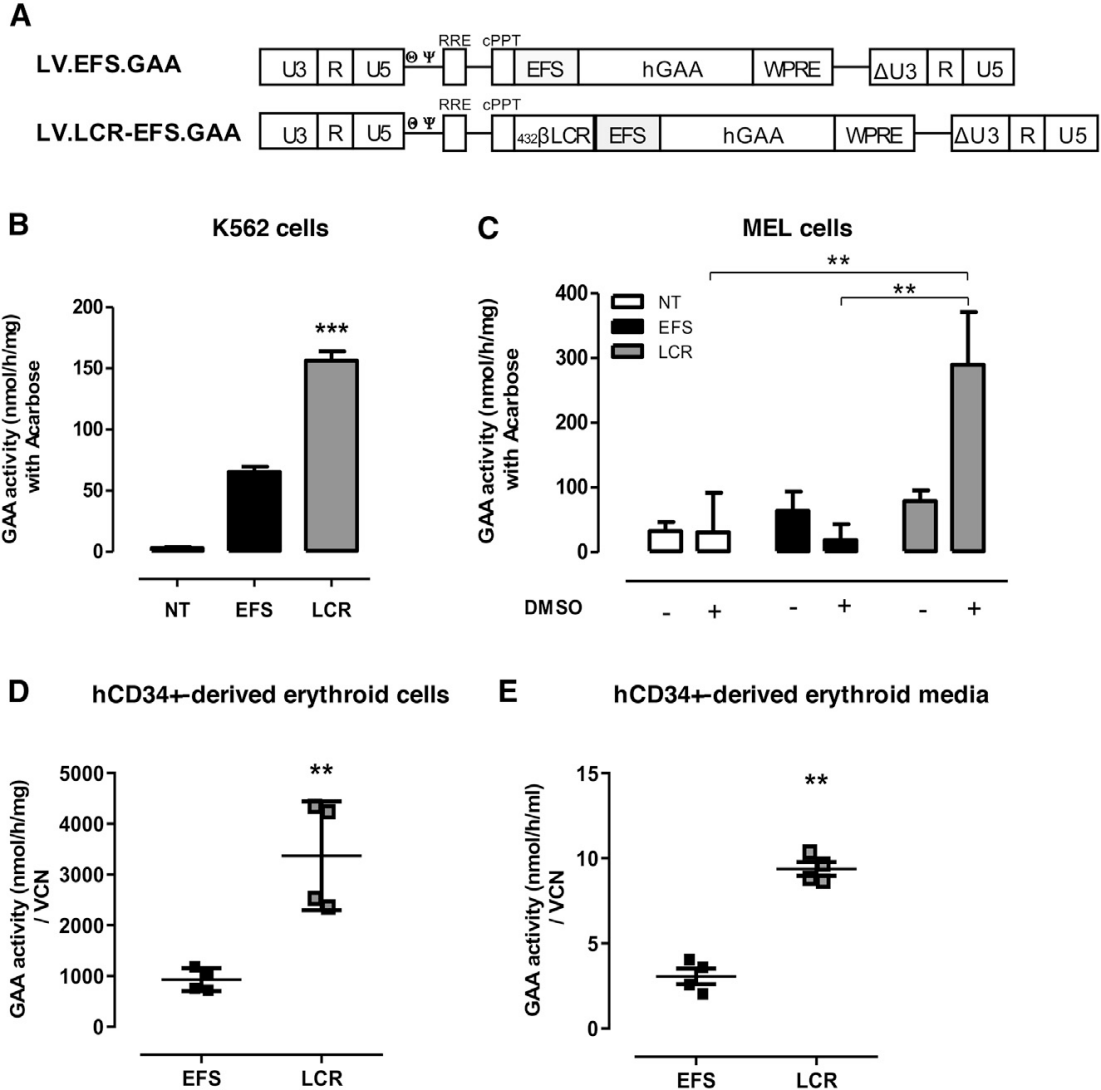
LV.LCR-EFS Vector Enhances GAA Expression in Erythrocyte-like Cells (A) Scheme of LV.EFS.GAA (EFS) and LV.LCR-EFS.GAA (LCR) used in the study. (B–D) GAA expression was measured as GAA specific activity in (B) K562 cells and (C) MEL cells, differentiated in erythrocyte-like cells in the presence of 2% DMSO, and (D) hCD34^+^ cells, differentiated in erythrocyte-like cells after transduction with the EFS or LCR vector. (E) GAA activity measured in conditioned media of hCD34^+^ cells differentiated in erythrocyte-like cells. K562 and MEL cells were transduced at an MOI of 5, and data represent the average of at least three independent experiments. hCD34 cells were transduced at an MOI of 50 prior to differentiation in erythrocyte-like cells *in vitro*, and data represent four independent experiments of two heathy donors. The data are shown as means ± SEM. ∗∗p < 0.01, ∗∗∗p < 0.001, by one-way ANOVA test.

We generated a self-inactivating LV with a pCCL backbone, which has been widely used in gene therapy clinical trials,^25^^,^^26^ as a therapeutic vehicle for the expression of a native form of hGAA in HSPCs and compared the GAA expression under the control of the EFS-1α promoter in the presence or absence of the human β-globin LCR enhancer, as previously described^43^ (Figure 1A; LV.LCR-EFS.GAA and LV.EFS.GAA, respectively). The presence of the LCR enhancer led to a 3-fold increase of GAA expression, measured by intracellular enzymatic activity, in K562 cells and a 6-fold increase in MEL cells, when differentiated into more mature erythroid cells in the presence of DMSO (Figures 1B and 1C, respectively). We next tested LV.LCR-EFS.GAA vector expression in human erythroid cells, which were differentiated *in vitro* from transduced hCD34^+^ cells of two healthy donors (HDs). Although the LV.LCR-EFS.GAA potency was donor-specific, we measured an average 3-fold increase of GAA expression in the LV.LCR-EFS.GAA-treated cells (Figure 1D) as well as in their growth media (Figure 1E) compared to the LV.EFS.GAA-treated hCD34^+^ cells or their media, suggesting that the activity of the LCR.EFS in erythroid cells leads to GAA overexpression and consequently GAA secretion.

### Tolerability of GAA Overexpression in hCD34^+^ Hematopoietic Stem Cells

**Figure 2:**
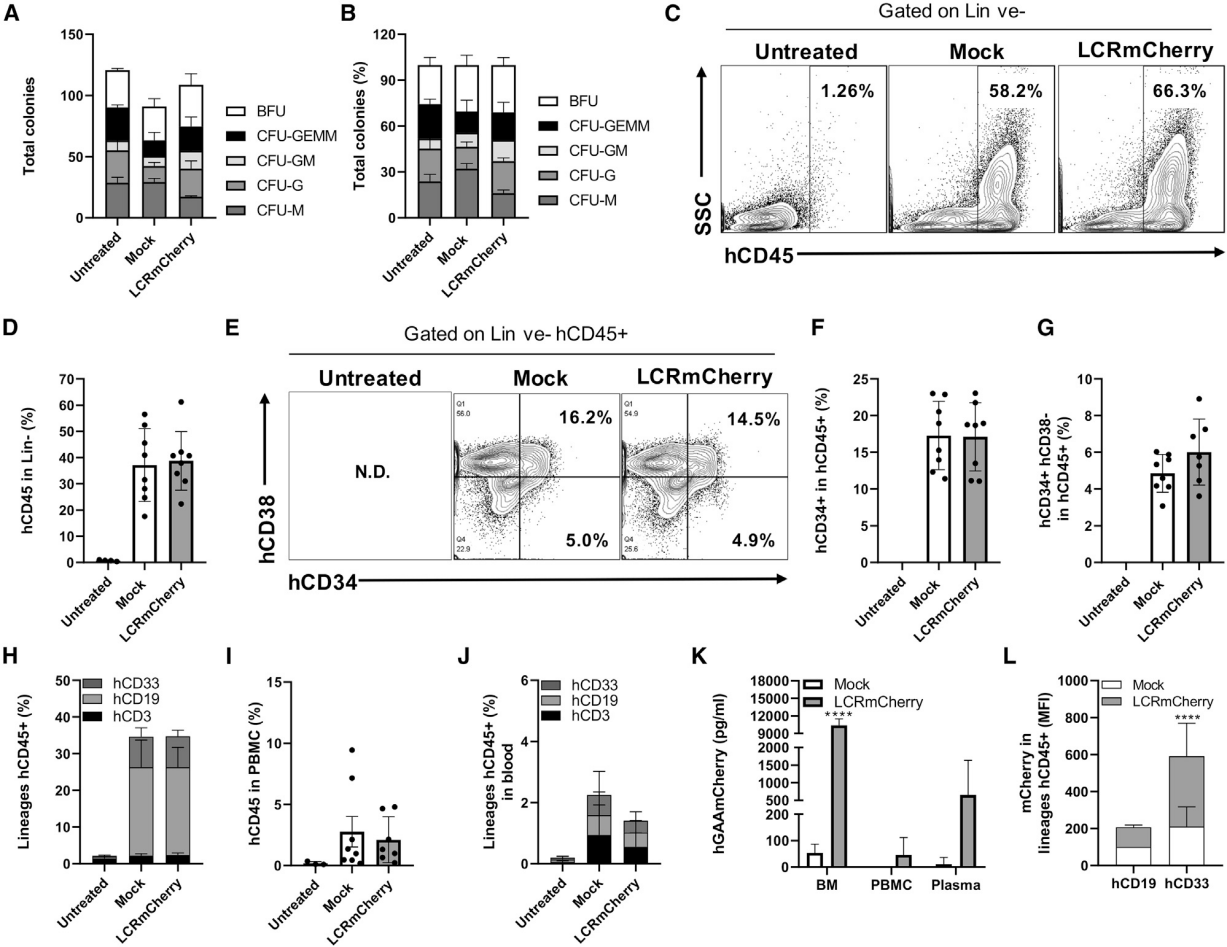
LV.LCR-EFS.GAA-Transduced hCD34^+^ Cells Differentiate and Engraft in NSG Mice Similarly to Mock-Transduced Cells Human CD34^+^ cells were transduced with LV.LCR-EFS.hGAAmCherry (LCRmCherry) or LV.EFS.eGFP (mock) at an MOI of 100 in the presence of LentiBOOST and protamine sulfate, and then kept in culture for a CFU assay or transplanted in NSG mice. (A) Number of differentiated colonies and (B) frequency distribution of total colony counts in mock- or LCRmCherry-transduced hCD34^+^ cells. Untreated group refers to healthy donor hCD34^+^ cells left unmanipulated as control group. NSG mice were sublethally irradiated to receive transduced hCD34^+^ cells (0.5 × 10^6^ cells/mouse; eight mice per group) via tail vein injection and culled 15 weeks later for analysis. Untreated refers to control NSG mice (total of four mice). (C and D) Representative fluorescence-activated cell sorting (FACS) plot of hCD45^+^ cells in bone marrow cells gated on live and lineage-negative (Lin-ve) cells (C) and their percentage distribution in untreated, mock, and LCRmCherry groups (D). (E–G) Representative FACS plot analysis of hCD34 hCD38 markers in bone marrow cells gated on Lin-ve hCD45^+^ cells (E) and the percentage distribution of hCD34^+^ (F) and hCD34^+^ hCD38^−^ (G) cells within Lin-ve hCD45^+^ cells per each group. (H) Percentage of hCD3^+^, hCD19^+^, and hCD33^+^ cells within hCD45^+^ bone marrow cells. (I and J) Percentage distribution of hCD45^+^ cells in PBMCs (I) and percentage of hCD3^+^, hCD19^+^, and hCD33^+^ cells in blood (J). (K and L) hGAAmCherry expression was detected in (K) bone marrow, PBMCs, and plasma by ELISA and (L) in hCD19^+^ and hCD33^+^ hCD45^+^ bone marrow cells by flow cytometry. The data are shown as means ± SD. ∗∗∗∗p < 0.0001, by by two-way ANOVA test, Sidak’s multiple comparison test.

To ensure that the overexpression of hGAA in the HSPCs does not lead to any adverse effects on HSPC function and capability, we investigated the engraftment capacity of LV.LCR-EFS.GAA-transduced hCD34^+^ cells from a third HD in NSG mice. For ease of detection, we generated an LCR.EFS vector carrying hGAA cDNA fused to mCherry (GAAmCherry). The GAAmCherry fused protein showed (1) similar GAA enzymatic activity and (2) *in vitro* cell uptake as the native form of hGAA (Figures S1B and S1C), and, in addition, (3) the mechanism of entry was through the M6P receptor (Figure S1D). We transduced hCD34^+^ cells from an HD with either the LV.LCR-EFS.GAAmCherry or a mock vector expressing GFP (LV.EFS.GFP) at an MOI of 100 using LentiBOOST and protamine sulfate to enhance transduction efficiency.^44^ As shown by total colony number and frequency of lineage-committed colonies (Figures 2A and 2B), the differentiation capacity of hCD34^+^ HSPCs modified with either the LV.LCR-EFS-GAAmCherry or mock vector remained unaltered when compared to untransduced cells. We observed that about 50% of colonies expressed GFP with 15 viral copy numbers (VCN)/cell while 28% of colonies expressed GAAmCherry with 2.4 VCN/cell; overall, we measured a 2-fold increased expression of GAA in LV.LCR-EFS.GAAmCherry-transduced hCD34^+^ cells compared to mock-transduced hCD34^+^ cells after 10 days in culture (Figures S2A and S2B).

The LV.LCR-EFS.GAAmCherry- or mock-transduced hCD34^+^ cells were introduced into NSG mice and we measured the engraftment of human cells at 15 weeks post-transplant. All recipient mice in both groups showed a similar percentage of hCD45^+^ cells in all hematopoietic organs. In bone marrow, we found on average 37.15% ± 13.85% and 38.74% ± 11.18% hCD45^+^ cells when gated on lineage-negative (Lin-ve) cells in both mock and LV.LCR-EFS.GAAmCherry groups, respectively (Figures 2C and 2D). In both groups, approximately 17% ± 4.6% of those cells were hCD34^+^ and 5.5% ± 1.4% were hCD34^+^ hCD38^−^ (Figures 2E–2G), demonstrating the engraftment of cells with a human HSPC immunophenotype. The engrafted cells were able to differentiate *in vivo* with no difference between mock- and LV.LCR-EFS.GAAmCherry-treated groups. In particular, 2% ± 0.6% hCD3^+^ (T lineage), 8.4% ± 2% hCD33^+^ (myeloid lineage) and 24% ± 6.4% hCD19^+^ (B lineage) cells were detected in bone marrow in both groups (Figure 2H). In blood, we detected on average 2.5% ± 2.7% hCD45^+^ cells in the transplanted mice and similar distribution of CD3^+^, CD19^+^, and CD33^+^ cells regardless of the vector used (Figures 2I and 2J). We measured the expression of GAAmCherry and, consistent with the vector design, we detected greater GAA overexpression in bone marrow compared to peripheral blood mononuclear cells (PBMCs) and the presence of marked protein in plasma. Consistently, GAAmCherry was 3-fold higher in hCD33^+^ cells compared to hCD19^+^ cells in the LCR-EFS-treated group (Figures 2J and 2K). Overall, these data suggest that the overexpression of GAA in hCD34^+^ cells through LV transduction does not affect their capacity to engraft or their ability to generate gene-modified progeny.

### Efficacy Study *In Vivo*: Rescue of the Clinical Phenotype and Reduction of Muscle Pathology

**Figure 3:**
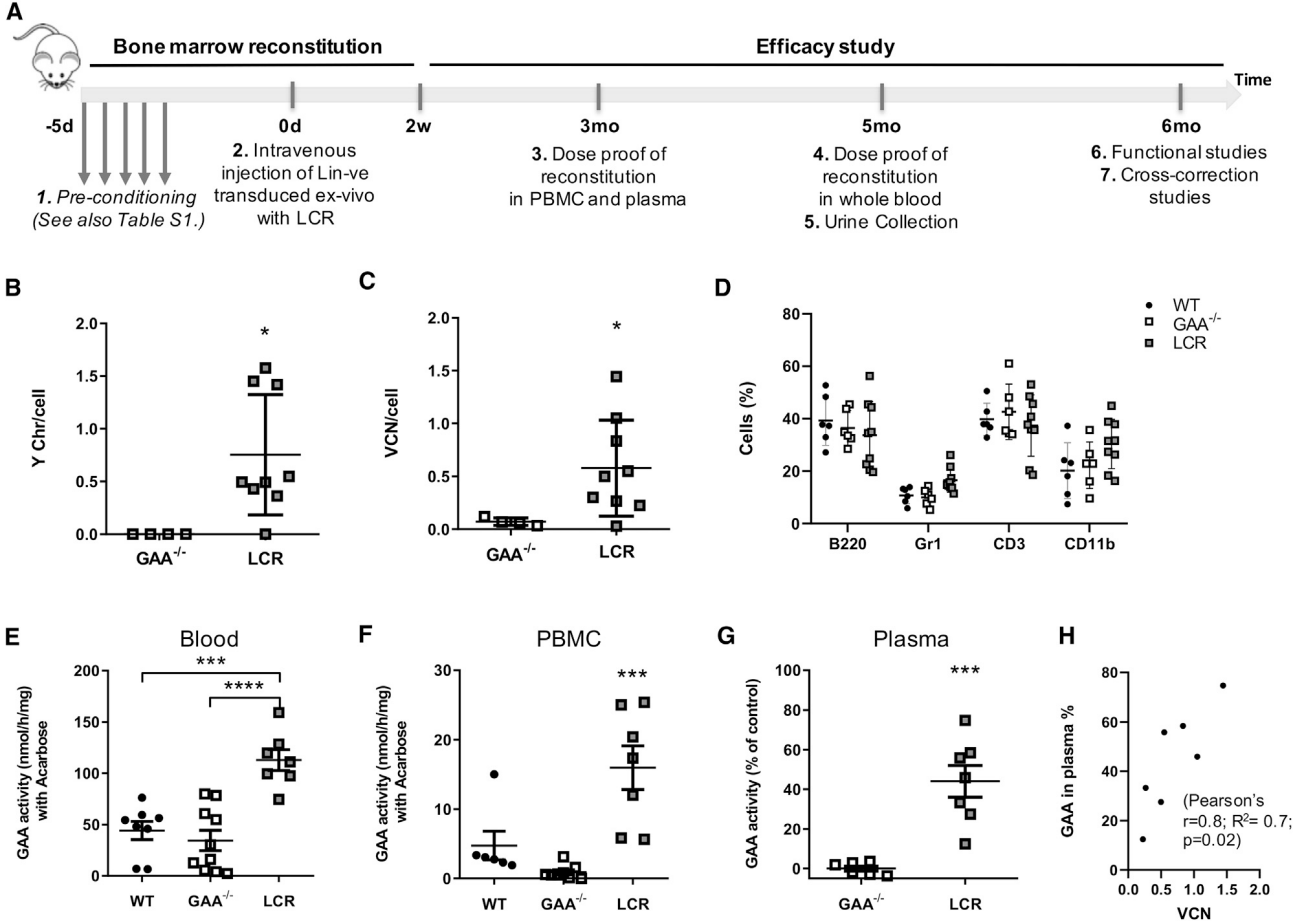
LV.LCR-EFS.GAA HSC Gene Therapy in a Murine Model of Pompe Disease Lin-ve cells from male GAA^−/−^ mice were transduced *ex vivo* with LV.LCR-EFS.GAA (LCR) and transplanted into conditioned GAA^−/−^ female mice. (A) Scheme of the experimental design, which illustrates the main steps and time points for the bone marrow reconstitution and efficacy study. (B and C) Engraftment (B) and VCN/cell (C) were measured in PBMCs at 12 weeks post-transplant. (D) Percentage of blood cells marked for B220, Gr1, CD3, or CD11b in control wild-type (●), control GAA−/− (empty square), and treated mice (gray-filled square) at 12 weeks after transplant. (E–G) The GAA expression was measured as GAA specific activity in (E) whole blood, (F) PBMCs, and (G) plasma, in which it is expressed as the percentage of increase compared to the GAA^−/−^ control. (H) Pearson correlation analysis between GAA activity in plasma and VCN/cell detected in PBMCs of treated mice at 12 weeks after transplant (r = 0.8, R^2^ = 0.7, ∗p = 0.02). Data are shown as means ± SEM of three independent experiments for a total of n = 6–10 mice per group.

To test the efficacy of the LV.LCR-EFS.GAA vector, we conducted studies in an established knockout model of Pompe disease (GAA^−/−^),^43^ which recapitulates the muscle defects associated with the condition. We transplanted 6-week-old female GAA^−/−^ mice with LV.LCR-EFS.GAA-modified male GAA^−/−^ HSPCs after a myeloablative conditioning regime (Figure 3A and details in Table S1). The transplanted animals showed recovery of blood cell lineages similarly to control GAA^−/−^ and wild-type animals. On average, the VCN in blood of recipient mice was 0.6 VCN/cell, ranging from 0.36 to 1.44 copies per cell, and engraftment of donor cells was assessed by Y chromosome qPCR between 36% and 100% in blood, except for one animal, which did not show any engrafted cells (Figures 3B–3D). Mice with gene-corrected cells showed a statistically significant increased level of GAA activity in whole blood and PBMCs when compared to knockout and wild-type mice (Figures 3E and 3F) and a consequent 44% increase in GAA activity in plasma, which was positively correlated with VCN/cell in PBMCs (Figures 3G and 3H; r = 0.8, p ≤ 0.02, n = 7, with R^2^ = 0.7).

**Figure 4:**
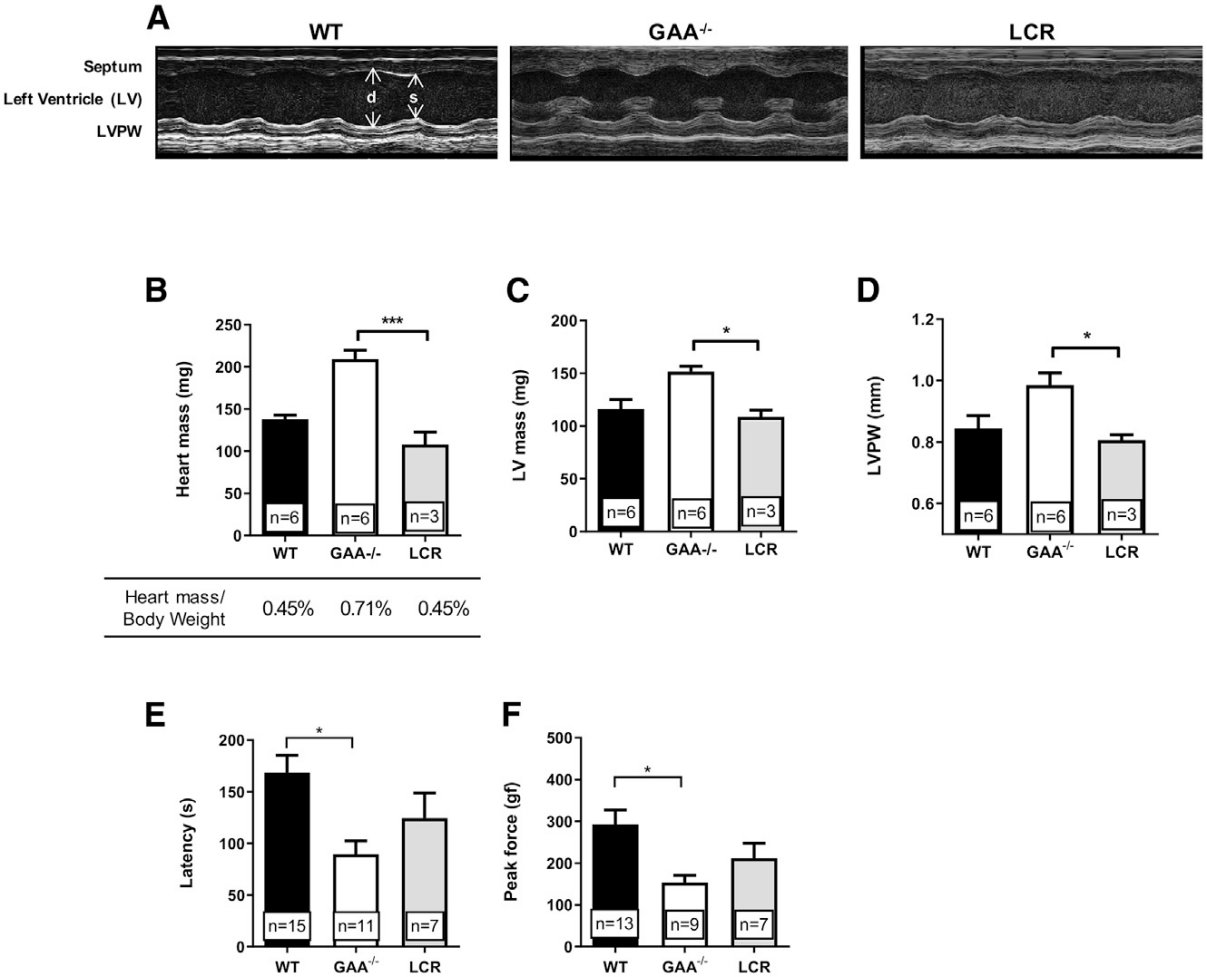
Assessment of Cardiac and Motor Functions in GAA^−/−^ Mice Treated with LV.LCR-EFS.GAA HSC Gene Therapy (A) Representative M-mode images showing septum, left ventricular space, and left ventricular posterior wall (LVPW) of wild-type, GAA^−/−^, and LV.LCR-EFS.GAA (LCR)-treated mice during diastole (d) and systole (s). (B–D) Heart mass and percentage of heart mass on total body weight (B), left ventricle (LV) mass (C), and thickness (D) of the LVPW of LCR-treated mice compared to wild-type and GAA^−/−^ littermates. (E and F) Motor coordination assessed by rotarod (E) and muscle strength (F) measured by grip strength meter of LCR-treated mice compared to wild-type and GAA^−/−^ littermates. Cardiac performances were evaluated via echocardiography in mice that showed VCN > 0.8 (details in Table S1). Data are shown as means ± SEM of three independent experiments with number of mice (n =) indicated in the graphs.

To determine the success of hGAA hematopoietic cell expression in the cross-correction of muscle tissues, we investigated the phenotypic effect of the gene therapy after 6 months post-transplant. We performed echocardiography and motor function tests to establish the recovery of functions previously reported as defective in GAA^−/−^ mice. The thickness of the heart muscle walls, clearly visible in untreated GAA^−/−^ animals, fully reverted upon gene therapy with LV.LCR-EFS.GAA back to wild-type dimensions (Figure 4A). Whole heart mass, left ventricular mass, and thickness of the left ventricular posterior wall in LV.LCR-EFS.GAA-treated mice were significantly reduced compared to untreated GAA^−/−^ mice and were comparable to control wild-type mice (Figures 4B–4D). The heart mass reduction was not influenced by the total body weight of the animals (Figure 4B). In addition to the cardiac dimensions, LV.LCR-EFS.GAA-treated mice improved several other heart parameters, including filling velocities and cardiac output, resembling wild-type performance (Table 1). Altogether, these data showed that lentiviral HSPC gene therapy rescued the hypertrophic cardiomyopathy typical of the disease.

**Table 1:** Cardiac Functions of LV.LCR-EFS.hGAA-Treated Mice Compared to Wild-type or GAA^−/−^ Control Group

Parameters	Trend of Transplanted Animals Compared to	Statistical Significance
Cardiac output (mL/min)	WT = similar; GAA^−/−^ = lower	p = 0.59
Ejection fraction (%)	WT = similar; GAA^−/−^ = similar	p = 0.61
Fractional area shortening	WT = higher; GAA^−/−^ = lower	p = 0.40
Fractional shortening (%)	WT = lower; GAA^−/−^ = lower	p = 0.08
Stroke volume (μL)	WT = similar; GAA^−/−^ = lower	p = 0.10
End diastolic volume (μL)	WT = similar; GAA^−/−^ = lower	p = 0.29
End systolic volume (μL)	WT = similar; GAA^−/−^ = similar	p = 0.79
Ascending aortic diameter (mm)^a^	WT = similar; GAA^−/−^ = lower	p = 0.12
Descending aortic diameter (mm)^a^	WT = similar; GAA^−/−^ = lower	p = 0.16
Transverse aortic diameter (mm)^a^	WT = similar; GAA^−/−^ = lower	p = 0.38
Ascending aortic velocity (mm/s)^a^	WT = higher; GAA^−/−^ = lower	p = 0.06
Descending aortic velocity (mm/s)^a^	WT = similar; GAA^−/−^ = lower	∗∗p = 0.0053
AV peak pressure^a^	WT = higher; GAA^−/−^ = lower	p = 0.09
Early ventricular filling velocity (mm/s)	WT = similar; GAA^−/−^ = lower	p = 0.13
Late ventricular filling velocity (mm/s)	WT = lower; GAA^−/−^ = lower	p = 0.34
E/A	WT = higher; GAA^−/−^ = similar	p = 0.37
LV mass	WT = similar; GAA^−/−^ = lower	∗p = 0.014
Aortic root	WT = similar; GAA^−/−^ = lower	p = 0.13
LV posterior wall thickness (mm) diastole	WT = similar ; GAA^−/−^ = lower	∗p = 0.022
Interventricular septum (mm) diastole	WT = similar ; GAA^−/−^ = similar/lower	p = 0.51
LV internal dimension (mm) diastole	WT = similar ; GAA^−/−^ = similar	p = 0.83

E/A, E wave/A wave; LV, left ventricle.

a Missing value

GAA^−/−^ mice showed significant skeletal muscle defects when assessed by rotarod and grip strength tests. Although not statistically significant, LV.LCR-EFS.GAA-treated mice showed increased running time and grip force compared to untreated GAA^−/−^ mice, respectively, suggesting improved motor coordination and muscle strength upon treatment (Figures 4E and 4F).

**Figure 5:**
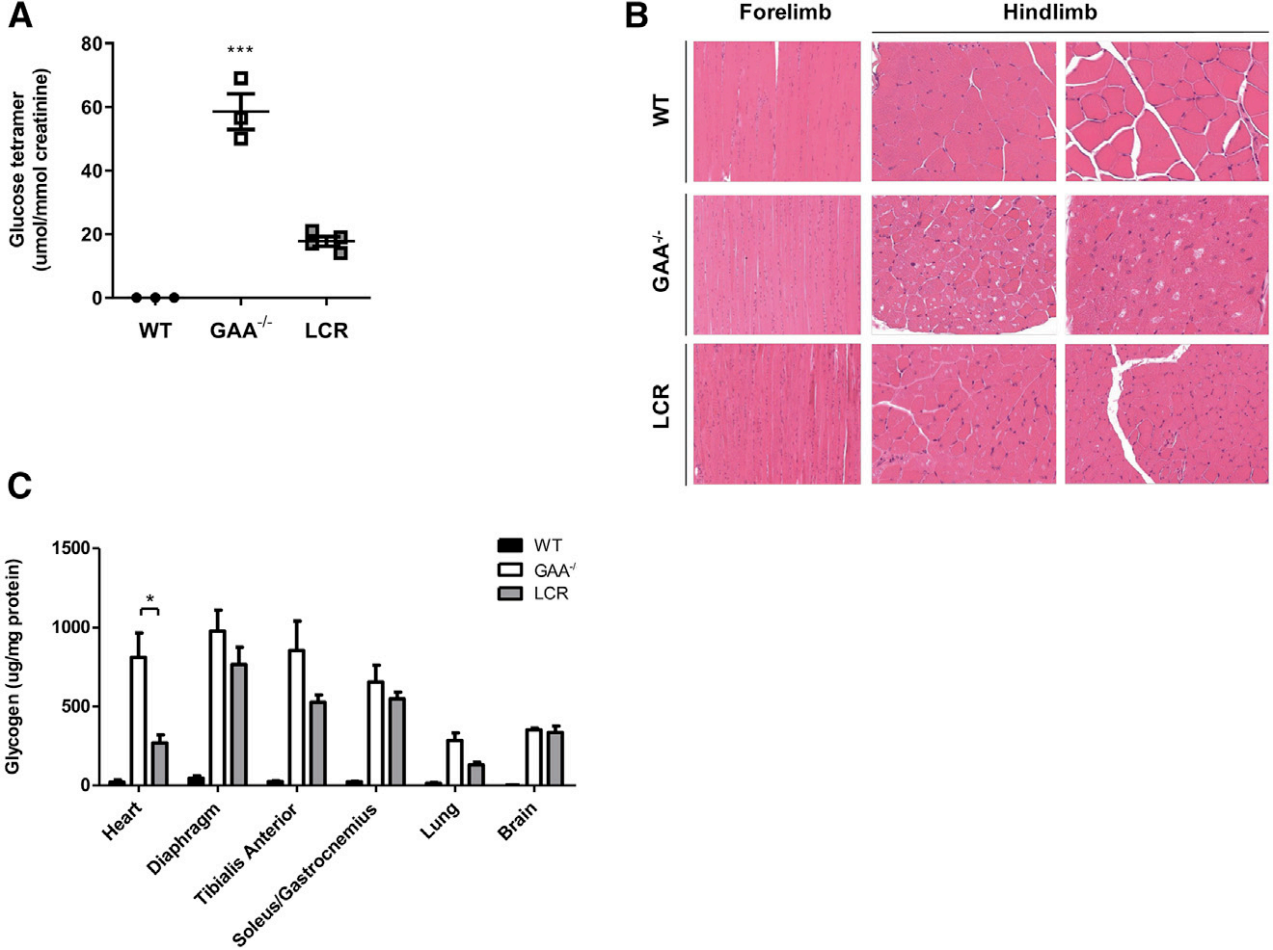
Assessment of Pathology in GAA^−/−^ Mice Treated with LV.LCR-EFS.GAA HSC Gene Therapy (A) Concentration of glucose tetramer detected in urine of wild-type, GAA^−/−^, and LV.LCR-EFS.GAA (LCR)-treated mice. (B) Representative H&E sections of whole forelimbs and hindlimbs of wild-type, GAA^−/−^, and LCR-treated mice showing a degree of fiber vacuolization. (C) Glycogen content measured in heart, diaphragm, tibialis anterior, soleus/gastrocnemius, lung, and brain homogenates of wild-type (black bar), GAA^−/−^ (white bar), and LCR-treated (gray bar) mice. Data are shown as means ± SEM of two independent experiments with n = 3–6 mice per group.

Analysis of skeletal muscles, heart, and lung showed hGAA protein in their homogenates, as detected by western blot (Figure S3). The GAA activity in all tissues but brain of LV.LCR-EFS.GAA-treated mice significantly increased compared to untreated GAA^−/−^ mice, but values were below the enzymatic activity detectable in wild-type mice (Figure S3A). In urine, the glucose tetramer, an established disease biomarker, was 4-fold lower in the LV.LCR-EFS.GAA-treated mice compared to untreated GAA^−/−^ mice (Figure 5A). The H&E staining of the whole forelimbs and hindlimbs showed a reduction of vacuole size and number of vacuolated muscle fibers in LV.LCR-EFS.GAA-treated mice compared to untreated GAA^−/−^ mice (Figure 5B). We also showed that the glycogen reduction correlated with increased GAA activity in tissues (Figure S3B). The most significant reduction of glycogen was measured in the cardiac muscles (66.6% reduction) followed by lung (54.4%), tibialis anterior (38.4%), diaphragm (21.7%), and soleus/gastrocnemius (16.2%), while the glycogen content detected in the brain compared to untreated GAA^−/−^ mice remained relatively unchanged (Figure 5C; Table S2).

The histological analysis of heart and diaphragm confirmed the biochemical measurements. The heart from LV.LCR-EFS.GAA-treated mice showed similar periodic acid-Schiff (PAS) and diastase-PAS (DPAS) staining to wild-type tissues. In diaphragm, we observed a general reduction of positive PAS-stained fibers, but some fibers still presented punctuated staining, indicating a residual accumulation of glycogen (Figure S4). Similarly, we observed a reduction of vacuolated fibers in H&E-stained sections as well as a reduction of acid phosphatase, a biomarker of skeletal muscle pathology, in heart and diaphragm of LV.LCR-EFS.GAA-treated mice compared to untreated GAA^−/−^ mice (Figure S5).

Altogether, these data indicate a systemic biochemical correction and an overall improved function in specific tissues upon lentiviral HSPC gene therapy compared to untreated GAA^−/−^ mice.

## Discussion

We designed a clinically relevant LV to be used in an HSPC gene therapy protocol for Pompe disease. Our vector contains a codon-optimized version of the native *GAA* gene under the transcriptional control of the ubiquitous mammalian EFS-1α promoter and the three active elements of the locus control region of the β-globin chain, namely H4, H3, and H2 (βLCR), both already in use in gene therapy clinical trials (ClinicalTrials.gov: NCT01306019, NCT01380990, NCT03311503, NCT03601286, NCT02151526, NCT01745120, and NCT03207009). The combination of EFS-1α and βLCR allows the expression of the transgene throughout the progeny of HSPCs with specific upregulation of expression in the erythrocyte lineage, both *in vitro* and *in vivo*.^43^ In contrast to other studies on HSPC gene therapy for Pompe disease, which used internal viral promoter prone to epigenetic silencing,^45^ this vector uses a mammalian stable promoter and a human LCR and has the potential for onward clinical translation.

This vector consistently showed the ability to enhance GAA expression and its intracellular as well as extracellular enzymatic activity in erythroid-like cell lines and in hCD34^+^ cells upon both *in vitro* and *in vivo* differentiation. LV.LCR-EFS.GAA-modified hCD34^+^ did not show any sign of toxicity, indicating that the vector design does not interfere with the stem cell capability, homing, and differentiation abilities. We did not observe any sign of genotoxicity induced by the overexpression of GAA in blood cells and specifically in erythrocytes. This is in line with previous data using encapsulated high doses of GAA in erythrocytes that showed that GAA concentrations up to 600 μg/mL are well tolerated and not deleterious for erythrocyte viability or function.^46^

By exploiting the HSPCs and their progeny as a biological factory of hGAA, we promoted cross-correction of GAA function through systemic release of hGAA by blood cells. Following a clinically relevant gene therapy protocol, which includes chemotherapeutic conditioning with busulfan and transplantation of gene-corrected HSPCs at a relevant young age, we demonstrated rescue of heart morphology and function as well as improved motor function and muscle strength up to 6 months after treatment, with similar outcomes having been shown by van Til et al.^42^ We also measured an effective albeit incomplete clearance of pathological biomarkers such as glucose tetramer in urine and glycogen in tissues, with the exception of brain tissues.

The use of busulfan as a conditioning agent and HSPC gene therapy have the unique potential to repopulate the CNS with gene-corrected microglial-like cells and release the therapeutic enzyme directly for neuronal uptake.^47^^,^^48^ However, the activity of hGAA detected in brain tissues in this study was poor and insufficient to reduce glycogen storage in this compartment. Data from HSPC gene therapy studies in other lysosomal storage disorders suggest that supranormal expression and secretion of GAA are required for CNS correction. Studies in MLD, MPS-1, and MPS-IIIA murine models all clearly showed that overexpression of the therapeutic gene was necessary for full biochemical, neuropathological, and behavioral correction. Overexpression was achieved either through the use of a high VCN in gene-corrected HSPCs and/or in combination with a myeloid-specific promoter driving increased expression in microglial cells.^35^^,^^39^^,^^40^^,^^48^ Notably, a recent work from Stok et al.^49^ showed GAA-positive microglia cells, an increase of GAA activity, and a reduction close to normal levels of glycogen in brain of GAA^−/−^ mice after lentiviral HSPC gene therapy with a codon-optimized variant of the native *GAA* gene, the expression of which was driven by a spleen focus-forming virus (SFFV) promoter. The combination of strong gene expression, through codon optimization of the gene and strong viral promoter, and high potency of integration (VCN around 7) clearly helped the therapeutic efficacy thoroughly. In our study we used a codon-optimized GAA sequence, but we only achieved a physiological VCN/cell of approximately 1 in HSPCs, which was ultimately suboptimal to restore GAA enzymatic activity at wild-type values in target tissues. In view of these data, the increase of VCN and therefore of GAA expression in donor-derived cells, including the myeloid progeny, would likely benefit the overall cross-correction, including the neuropathology associated with Pompe disease. Due to the size of the LV.βLCR-EFS-1α.hGAA vector (about 13 kb in length), however, the achievement of such a therapeutic window of copy numbers can be limited by the transduction efficiency in HSPCs.^50^^,^^51^ We have recognized the importance of vector design for the safety and success of a clinical trial, and are currently looking at the use of constitutive promoters, such as phosphoglycerol kinase (PGK) promoter, to drive GAA expression broadly in all hematopoietic lineages and allow higher transduction efficiency and improved disease correction.^51^, ^52^, ^53^, ^54^, ^55^ A few studies compared PGK promoter to myeloid-restricted promoters for therapeutic efficacy in models of inherited metabolic diseases and clearly showed the potential of transgene expression driven by PGK promoter in lentiviral gene therapy, with notably high expression of transgene in brain at a relatively low VCN.^40^^,^^56^

In addition to *GAA* gene expression, two other molecular aspects play a crucial role in the potential success of HSPC gene therapy for Pompe disease: (1) GAA secretion from corrected cells and (2) GAA uptake in target tissues. In normal individuals, only a relatively low level of physiological GAA precursor is secreted in the extracellular space.^57^ Although the *GAA* overexpression in modified cells leads into an increase of hGAA release in the milieu, the implementation of an enhanced secretion strategy, e.g., using signal peptide modification of a transgene, may help to increase the release efficiency and therefore secrete greater amounts of GAA at lower or equivalent VCN/genome.^22^ However, as several studies on ERT for Pompe disease report,^3^^,^^13^^,^^58^^,^^59^ increased bioavailability of hGAA in plasma does not by itself necessarily lead to complete reversion of primary and secondary defects in the diseased muscles since the other important factor to consider is the efficiency of uptake into muscle cells. In the context of systemic AAV gene therapy, it was clearly shown that the intramuscular and intrahepatic expression, but not only the restricted liver-specific expression, of a secretable form of hGAA was able to achieve full correction of pathology in muscles.^23^ Therefore, regardless of whether the rhGAA is provided in the plasma by ERT or AAV liver-specific secretion or HSPC gene therapy, there is a need for the enzyme to be efficiently taken up by the skeletal muscles to perform its glycogen clearance activity. A possible limitation is the heterogeneous expression profile of the cation-independent mannose 6-phosphate receptor (CI-M6PR, also known as insulin-like growth factor 2 receptor [IGF2R]), which acts as a unique cellular door for lysosomal enzymes, in muscle tissues. Modification of rhGAA by chemical conjugation with synthetic oligosaccharides carrying M6P showed improved affinity to CI-M6PR and therefore efficacy of the cross-correction in the animal model of Pompe disease.^60^^,^^61^ Alternatively, a design of a novel fused protein between GAA and IGF2 (IGF2-GAA) had also shown improved uptake in skeletal muscles through a glycosylation-independent lysosomal targeting pathway.^62^ Other attempts to enhance CI-M6PR-independent tissue uptake focused on the generation of chimeric forms of rhGAA containing a humanized antibody fragment against an anti-DNA antibody, 3E10 antibody.^63^ Hence, strategies to enhance GAA uptake in target tissues are highly desirable.

Treatments aimed to target multisystem diseases are a big challenge due to the need for wide distribution and efficient uptake into tissues. As an alternative to currently available ERT, *in vivo* AAV gene therapies directed to transgene expression in liver and/or in muscle have been proven successful in an animal model of Pompe disease and are currently tested in phase I/II clinical trials for LOPD (ClinicalTrials.gov: NCT02240407 and NCT03533673). However, these AAV gene therapy approaches would not address the correction of the neuropathology associated with Pompe disease, due to the presence of the blood-brain barrier that shields the CNS from proteins found in the blood circulation. In addition, the AAV gene therapy clinical trials have restrictions on the selection of pediatric patients into the treatment due to the potential durability of the vector and transgene expression in regenerating tissues, which, as a consequence, may require a second dosage of AAV gene therapy to maintain the therapeutic efficiency and therefore pose concerns regarding immune responses.^64^ Alternatively, HSPC lentiviral gene therapy gives the opportunity of a definitive long-lasting potentially curative procedure, which is also able to address neurological defects through a single-dose administration and avoid immunological effects, which can be suitable both for IOPD and LOPD. This study has demonstrated the use of a clinically applicable LV candidate for the treatment of Pompe disease and that expression of hGAA in HSPCs does not show adverse effects on hCD34^+^ engraftment and differentiation. This strategy showed biochemical improvements and correction of a number of the phenotypes associated with the disease. However, it is clear that there are limitations to the current vector/transgene design that need to be addressed to allow complete glycogen clearance and full phenotypic correction. By implementing our vector to highly express codon-optimized GAA through the increase of transduction efficiency and the use, eventually, of an alternative strong promoter as PGK, we might achieve that therapeutic threshold, set high in Pompe disease, needed to observe normalization of glycogen in tissues but without compromising safety. Further studies are underway to potentiate expression and improve uptake in skeletal muscles and CNS.

## Materials and Methods

### Vector Cloning Strategy and Lentiviral Production

An hGAA cDNA sequence was codon optimized and commercially synthesized by GenScript Biotech. This sequence was cloned as an *Afe*I/*Sal*I fragment into pCCLsincppt-EFS1α-ADA-WPRE or pCCLsincppt-β-LCR (HS4,3,2)-EFS1α-ADA-WPRE, substituting the ADA transgene. To generate hGAAmCherry, we subcloned hGAA cDNA in pCR2.1 TOPO plasmid and cloned in-frame mCherry cDNA at the 3′ end by Gibson assembly (New England Biolabs, 2016). A spacer of eight Ala residues was designed between the two proteins. The fused cDNA transgene was cloned into LV backbone by standard cloning technique. Lentiviral particles were produced and titered as previously described in HEK293T cells, vesicular stomatitis virus glycoprotein G (VSV-G) pseudotyped.^43^ Quantification of viral particles was carried out by targeting the WPRE region in the LVs and murine titin or human β-actin genomic regions in cells by using a TaqMan real-time PCR assay.

### Cell Cultures

The K562 (human erythroleukemia) cell line was maintained in RPMI 1640 medium (Invitrogen, Paisley, UK) supplemented with 10% fetal bovine serum (FBS, Sigma-Aldrich, Poole, UK) and 10 μg/mL each of penicillin and streptomycin (pen/strep, Invitrogen), while MEL and HEK293T cells were maintained in 10% FBS and 1% pen/strep Dulbecco’s modified Eagle’s medium (DMEM, Invitrogen). MEL cells were differentiated for 4 days in the presence of 2% DMSO, and their differentiation was determined by morphological changes in flow cytometry (FCM).^43^ Cells were transduced at an MOI of 5 for 4 h and then maintained 3 days in culture before harvesting for biochemical analysis. An immortal myogenic cell line derived from *H-2K*^*b*^-tsA58 immortomouse (gift from Prof. Jennifer Morgan) was cultured as myoblasts and differentiated in myotubes as described in Muses et al.^65^ MS5 (mouse bone marrow stroma) cells were maintained in α-minimum essential medium (α-MEM) (Invitrogen) with 20% serum and 1% pen-strep (Invitrogen). hCD34^+^ cells were isolated from leukapheresis by magnetic-activated cell sorting (MACS) technology and kept in culture in serum-free StemSpam medium (STEMCELL Technologies, Grenoble, France) supplemented with 300 ng/mL human *fms*-related tyrosine kinase 3 (hFlt3), 300 ng/mL human stem cell factor (hSCF), 100 ng/mL human thyroperoxidase (hTPO), and 20 ng/mL human interleukin-3 (hIL-3) (all PeproTech, Rocky Hill, NJ, USA) for 16 h. These cells were then transduced at an MOI of 50 overnight and differentiated *in vitro* in erythrocytes following an adapted version of the protocol published by Giarratana et al.^66^ On day 18 after the start of differentiation, cells were harvested for biochemical and morphological analyses. Health donor hCD34^+^ cells were transduced at an MOI of 100 in the presence of 1mg/ml LentiBOOST and 4 μg/mL protamine sulfate in StemSpam containing 100 ng/mL hSCF, hFlt3, and hTPO each and 60 ng/mL hIL-3 for *in vivo* experiments. Informed written consent to use hCD34^+^ cells from HDs was obtained in accordance with the Declaration of Helsinki and the ethical approval from the Great Ormond Street Hospital for Children NHS Foundation Trust and the Institute of Child Health Research Ethics (08/H0713/87). Lin-ve cells were isolated from murine bone marrow and purified by negative selection through MACS technology (Miltenyi Biotec). Lin-ve cells were transduced at an MOI of 100 in StemSpam medium enriched with 100 ng/mL mSCF, 100 ng/mL mFlt3, 25 ng/mL hTPO (all PeproTech, Rocky Hill, NJ, USA), 2% FBS, and 1% pen/strep. Transduction efficiency was tested after 7 days of culture, changing media every other day. A colony-forming unit (CFU) assay was performed in MethoCult GF M3434 (STEMCELL Technologies, Grenoble, France). LSK^+^ cells were sorted as previously described.^67^ All cells were cultured at 37°C at 5% CO_2_.

### Experimental Animals and Bone Marrow Reconstitution

All animal studies were approved by the Institutional Research Ethics Committee (Great Ormond Street Institute of Child Health, University College London [UCL], UK) and performed according UK Home Office Animal Welfare Legislation. NSG mice (NOD.Cg-*Prkdc*^*scid*^ *Il2rg*^*tm1Wjl*^/SzJ) were supplied by the UCL breeding facility. GAA knockout mice (B6;129-*Gaa*^*tm1Rabn*^*/*J) were supplied by The Jackson Laboratory. Mice were housed in single ventilated cages, in pathogen-free conditions, and given *ad libitum* access to food and water. GAA^−/−^ mice were bred as heterozygous, and littermates were genotyped to identify homozygous and wild-type mice. Both female and male animals were used at 6–8 weeks of age. NSG mice were irradiated at 3 Gy for 90 s before bone marrow reconstitution with 0.5 × 10^6^ hCD34^+^ cells/mouse. Female GAA^−/−^ mice were either irradiated with 6 Gy + 4 Gy in 2 consecutive days or conditioned with intraperitoneal (i.p.) injections of 25 mg of busulfan/kg/day for 5 days (6 mg/mL Busilvex, Pierre Fabre). Bone marrow from male mice was flushed from femurs and tibia and processed for Lin-ve or LSK^+^ cell purification as detailed above. About 0.5–1.2 × 10^6^ cells/mouse were intravenously (i.v.) injected into pre-conditioned female recipient mice (for more details, see Table S1). Animals were starved the night before tissue harvesting to eliminate interference of cytoplasmic glycogen coming from food intake, especially in liver tissue. On the day of the culling, animals were exsanguinated via cardiac puncture and Dulbecco’s phosphate-buffered saline (D-PBS) perfused to remove all blood in circulation while under anesthesia with 1%–2% isoflurane in O_2_.

### Transduction Efficiency and Engraftment

Genomic DNA was extracted from cell pellets by using a DNeasy Blood and Tissue kit (QIAGEN, West Sussex, UK) following the manufacturer’s instructions. Before genomic extraction, pellets of Lin-ve cells were treated with Benzonase nuclease (Merck Millipore/Novagen, UK) for 30 min at room temperature (RT). Average VCN/cell was determined by WPRE RT-PCR, while engraftment was determined by Y chromosome copy/cell, against a standard curve, in Platinum Quantitative-PCR SuperMix-UDG (uracil-DNA glycosylase ) with ROX (Invitrogen, UK), as previously described.^43^

### FCM

Cells from spleen, thymus, bone marrow, and peripheral blood were freshly stained in D-PBS with 2 mM EDTA, 0.02% NaN_3_, and 0.5% BSA containing tissue-specific antibodies. For PBMC isolation, red cell lysis was performed with red blood cell (RBC) lysis buffer (BioLegend, San Diego, CA, USA), and the leukocyte populations were marked with murine (m)CD3 (eBioscience), B220 (BD Biosciences), mCD11b (BD Biosciences), or Gr1 (eBioscience). For xenotransplants, bone marrow cells were marked with human lineages (hLineages), hCD45, hCD34, and hCD38 or with hCD45, hCD3, hCD33, hCD19; PBMCs and splenocytes were stained for hCD45, hCD3, hCD33, and hCD19, while thymocytes were stained for hCD45, hCD3, hCD4, and hCD8 (all eBioscience). After 30 min at RT in the dark, cells were washed and re-suspended in 80 μL of D-PBS with 2% FBS, 0.02% NaN_3_, and 1% paraformaldehyde (PFA) for cytometry analysis. Events were acquired either by using a BD FACSCalibur platform (BD Biosciences) or Cytoflex platform (B53000, Beckman Coulter) and then analyzed by FlowJo v10.

### GAA Enzymatic Activity and Glycogen Assay

Cell pellets were lysed in 150 mM NaCl, 25 mM Trizma buffer at pH 6.4 containing 1% Triton X-100 and 1% protease inhibitor cocktail (all reagents from Sigma-Aldrich). Tissues were homogenized in D-PBS + 1% Triton X-100 and 2% protease inhibitor cocktail by using Precellys tubes containing ceramic beads and two cycles at 5,700 rpm, 30 s each, with intervals of 10 s in Precellys homogenizer. Cell debris was removed by centrifugation, and lysates were tested for GAA activity. Dried blood spots were eluted in water and analyzed by using a semi-automated protocol developed and validated at the department of Chemical Pathology Laboratory at Great Ormond Street Hospital (GOSH). The activity of GAA was measured by using the artificial substrate 4-methylumbelliferyl-α-d-glucopyranoside (4-MUG, Sigma), diluted in McIlvaine citrate buffer (pH 4.0), in the presence of 7.5 μM acarbose, which inhibits non-lysosomal α-glucosidase. The enzymatic reaction was stopped with 0.25 M glycine (pH 10.4), and the release of 4-MU was detected by spectrofluorimetry at 360 nm. The GAA activity was expressed as nmol of 4-MU released hourly per mg of protein or per mL of samples, for media and plasma. The glycogen content was determined by using the fluorimetric protocol of a glycogen assay kit (Sigma) and expressed as ng/mg of protein. Protein concentration was determined via a Pierce bicinchoninic acid (BCA) protein assay kit (Thermo Scientific).

### Western Blot

Tissue homogenates were loaded on NuPAGE 4%–12% Bis-Tris protein gels (Thermo Fisher Scientific, NP0321BOX) in denaturing condition for SDS-PAGE. Proteins were then transferred to a polyvinylidene fluoride (PVDF) membrane (Bio-Rad, 1620177), blocked for 1 h with 5% milk in PBS-T (PBS buffer containing 0.05% Tween 20), and then blotted either with anti-hGAA antibody (Abcam, ab137068) or anti-mGAPDH (Cell Signaling Technology, 2118S) as primary antibodies. Secondary goat polyclonal anti-immunoglobulin G (IgG) horseradish peroxidase (HRP)-conjugated antibodies (Invitrogen, G-21234) were detected by using SuperSignal West Pico PLUS chemiluminescent substrate (Thermo Fisher Scientific, 34579) and a Bio-Rad ChemiDoc XRS system.

### Glucose Tetramer in Urine

Urine was desalted using a mixed bed ion-exchange resin (Duolite MB-6113 resin) activated in 2 M acetic acid, and the supernatant was removed and an internal standard (cellotetraose) was added. Glucose tetramer (Glc4) was measured by high-performance liquid chromatography (HPLC) (anion-exchange chromatography) with electrochemical (pulsed amperometric) detection using the Thermo Scientific Dionex ICS-5000+ analyzer with a KOH eluent generation cartridge. The Glc4 was expressed as μmol/mmol creatinine; the analyses were performed in blinded experiments by the Chemical Pathology Laboratory at GOSH as described in Manwaring et al.^68^

### Assessment of Cardiac Function Using Ultrasound

Cardiac function was analyzed by using a VisualSonics Vevo 2100 system with an MS550D transducer (FujiFilm VisualSonics) in blinded experiments. Mice were anesthetized and maintained under 1%–2% isoflurane in O_2_. Electrocardiogram and respiration were monitored and hair was removed from the chest and neck using depilation cream. Parasternal long axis and basal, middle, and apical short axis views were acquired in B-mode in order to measure end diastolic, end systolic, and stroke volume, cardiac output, and ejection fraction using Simpson’s method. Fractional shortening, anterior and inferior wall thickness, was measured from short axis M-mode. Diastolic function was assessed by measurement of E wave/A wave (E/A) ratios using pulse wave Doppler in the apical 4 chamber view. Ascending and descending aortic velocities were measured using pulse wave Doppler in the aortic arch view, while left and right common carotid artery velocities were measured using pulse wave Doppler at the bifurcation of the artery.

### Assessment of Motor Functions

After a 20-min acclimation period in the behavioral room, mice were assessed for motor coordination by a rotarod for mice (47600, Ugo Basile) and for muscle strength by a grip strength meter for mice (47200, Ugo Basile). Mice from different experimental arms were equally randomized in groups and tested simultaneously to reduce day, time, or environmental interferences in the behavioral tests. In the rotarod, mice were trained at minimal rotation of 4 rpm for 5 min and then in accelerating rotation mode going from 4 to 40 rpm in 5 min, twice, with a 5-min break between trials. Then, the latency to fall from the rotarod was recorded and averaged from three additional runs in accelerating rotation mode, as above, with a 5-min break between runs. The maximum time given per run was 500 s. If animals reversed their direction during the run, they were replaced in the correct direction without stopping the run. The grip strength of forelimbs and hindlimbs was measured by placing the mice over a grasping grid and by pulling by their tail backward. The grasping grid was fitted to a force detector connected to a peak amplifier that automatically recorded the peak force; we reported the averaged peak force of three consecutive pulls. The pulling force was applied at constant rate through the help of quality control software, i.e., DCA software (38500-001, Ugo Basile).

### Histology

Murine tissues were OCT embedded and frozen in chilled isopentane using liquid nitrogen. Cryopreserved tissues were then sectioned at 10 μm on a cryostat (model CM1850, Leica Biosystems). The PAS/D-PAS, H&E, and staining on frozen tissues followed the methods published in Dubowitz et al.^69^ One whole forelimb and hindlimb per mouse were instead fixed in formalin, skinned, and inked to identify the dorsal/ventral orientation, decalcified, and paraffin embedded. Sections at 4 μm were cut through the limb, proximal to distal (shoulder to paw) and stained for H&E. All stained slides were scanned and captured by Leica SCN400, while images were viewed and captured by using the Leica Slidepath digital image hub application.

### Statistical Analysis

A Student’s t test or one-way ANOVA was used to determine statistically significant differences between mean values of two or more different datasets, followed by Bonferroni’s multiple comparison test unless otherwise described. Pearson’s r was used for correlation between values. All methods were run using GraphPad Prism v6 (GraphPad, La Jolla, CA, USA). The significance level is expressed as follows: ∗p < 0.05, ∗∗p < 0.01, ∗∗∗p < 0.001, ∗∗∗∗p < 0.0001.

## Author Contributions

Conceptualization, H.B.G., G.P., and C.M.-E.; Methodology, G.P. and C.M.-E.; Investigation, G.P., Y.-K.A.C., S.W., D.S., D.C., D.B., H.P., N.P., K.W., and M.C.; Resources, R.P., D.S., D.B., H.P., and D.L.-R.; Formal Analysis, Data Curation and Writing – Original Draft, G.P.; Visualization, G.P. and H.B.G.; Writing – Review & Editing, H.B.G.; A.J.T., S.B., and G.S.; Funding Acquisition, H.B.G. and A.J.T.; Supervision, R.P., G.S., A.J.T., and H.B.G.

## Conflicts of Interest

H.B.G. is currently employed by Orchard Therapeutics. A.J.T. is on the scientific advisory boards of Orchard Therapeutics and Rocket Pharmaceuticals. C.M.-E. and A.P.-G. are employed by GlaxoSmithKline plc group. The remaining authors declare no competing interests.

## Supplemental Information

Document S1. Figures S1–S5 and Tables S1 and S2

Document S2. Article plus Supplemental Information
