# Supplementary material for: Lentiviral Hematopoietic Stem Cell Gene Therapy Rescues Clinical Phenotypes in a Murine Model of Pompe Disease
Source: Mol Ther Methods Clin Dev. 2020 Jul 6;18:558–70. doi: 10.1016/j.omtm.2020.07.001 (PMC7396971; doi:10.1016/j.omtm.2020.07.001)
Supplement: Document S1. Figures S1–S5 and Tables S1 and S2 [file mmc1.pdf]

OMTM, Volume *18*

Supplemental Information

Lentiviral Hematopoietic Stem Cell Gene Therapy Rescues Clinical Phenotypes in a Murine Model of Pompe Disease

Giuseppa Piras, Claudia Montiel-Equihua, Yee-Ka Agnes Chan, Slawomir Wantuch, Daniel Stuckey, Derek Burke, Helen Prunty, Rahul Phadke, Darren Chambers, Armando Partida-Gaytan, Diego Leon-Rico, Neelam Panchal, Kathryn Whitmore, Miguel Calero, Sara Benedetti, Giorgia Santilli, Adrian J. Thrasher, and H. Bobby Gaspar

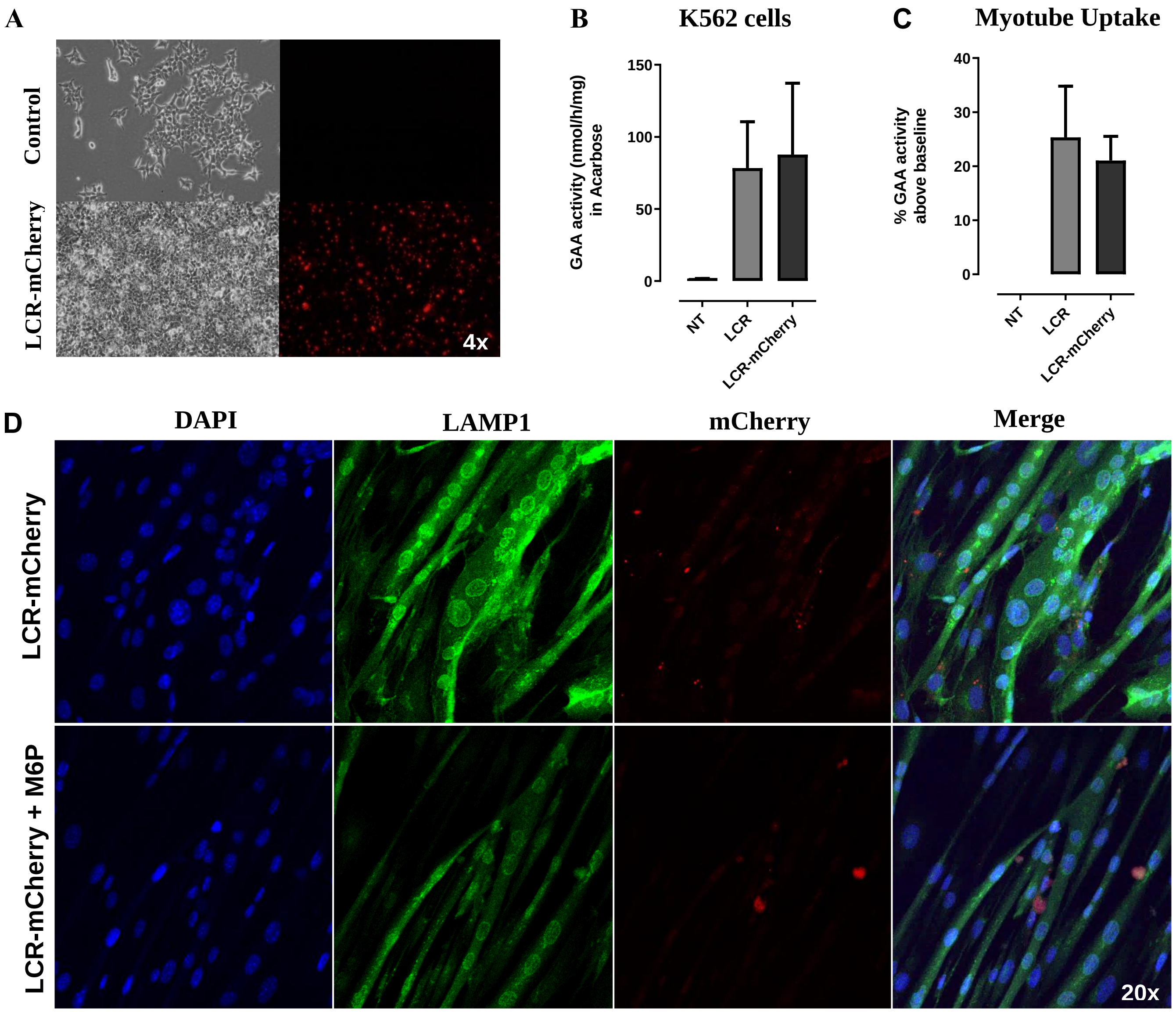

Figure S1. Evaluation of hGAAmCherry fusion protein. A) Representation of control 293T cells and packaging cells after transfection with LV.LCR-EFS.GAAmCherry (LCRmCherry). **B)** GAA activity measured in unmanipulated (NT) and transduced either with LV.LCR-EFS.GAA (LCR) or LCRmCherry K562 cells. **C)** Increase of GAA activity in murine myotube cells above baseline after exposure for 24 h to media conditioned for 3 days by unmanipulated K562 cells (NT), LCR or LCRmCherry transduced K562 cells. **D)** Representative H2K-2B4 myotubes images after exposure to 1uM GAAmCherry in presence or absence of 5mM M6P. Myotubes were stained for nuclei (DAPI), lysosomes (LAMP-1) and GAA (mCherry) detection. Data are shown as means ± SEM of 2 independent experiments.

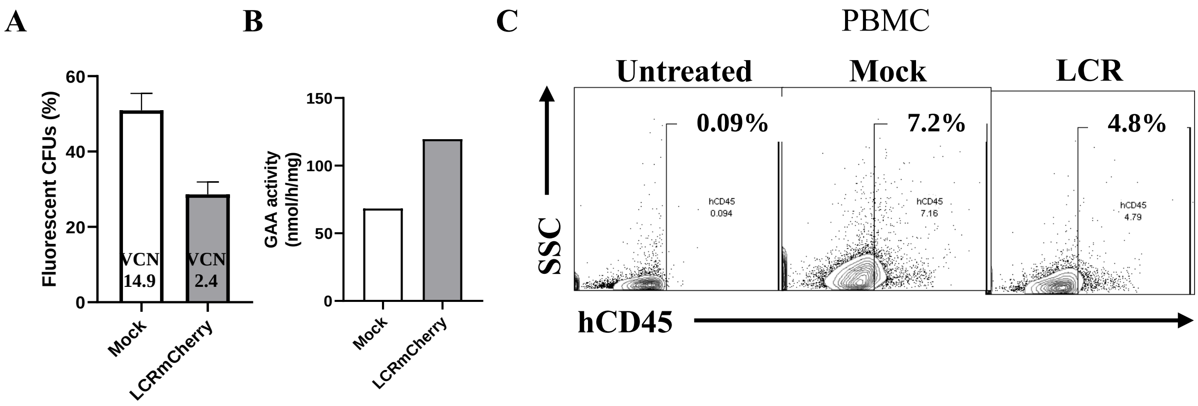

Figure S2. hCD34+ cells transduced with LV.LCR-EFS.GAAmCherry vector. A) Percentage of fluorescent positive colonies in the CFUs of Mock or LV.LCR-EFS.GAAmCherry (LCRmCherry) transduced hCD34+ cells. Data are shown as means ± SD. **B)** GAA activity measured in hCD34+ cells left in culture for 10 days after transduction with Mock or LCRmCherry vector. **C)** Representative FACS analysis of hCD45+ cells detected in PBMC of untreated (n=4), mock or LCRmCherry transplanted NSG mice (8 mice per group) at 15 weeks post-transplant.

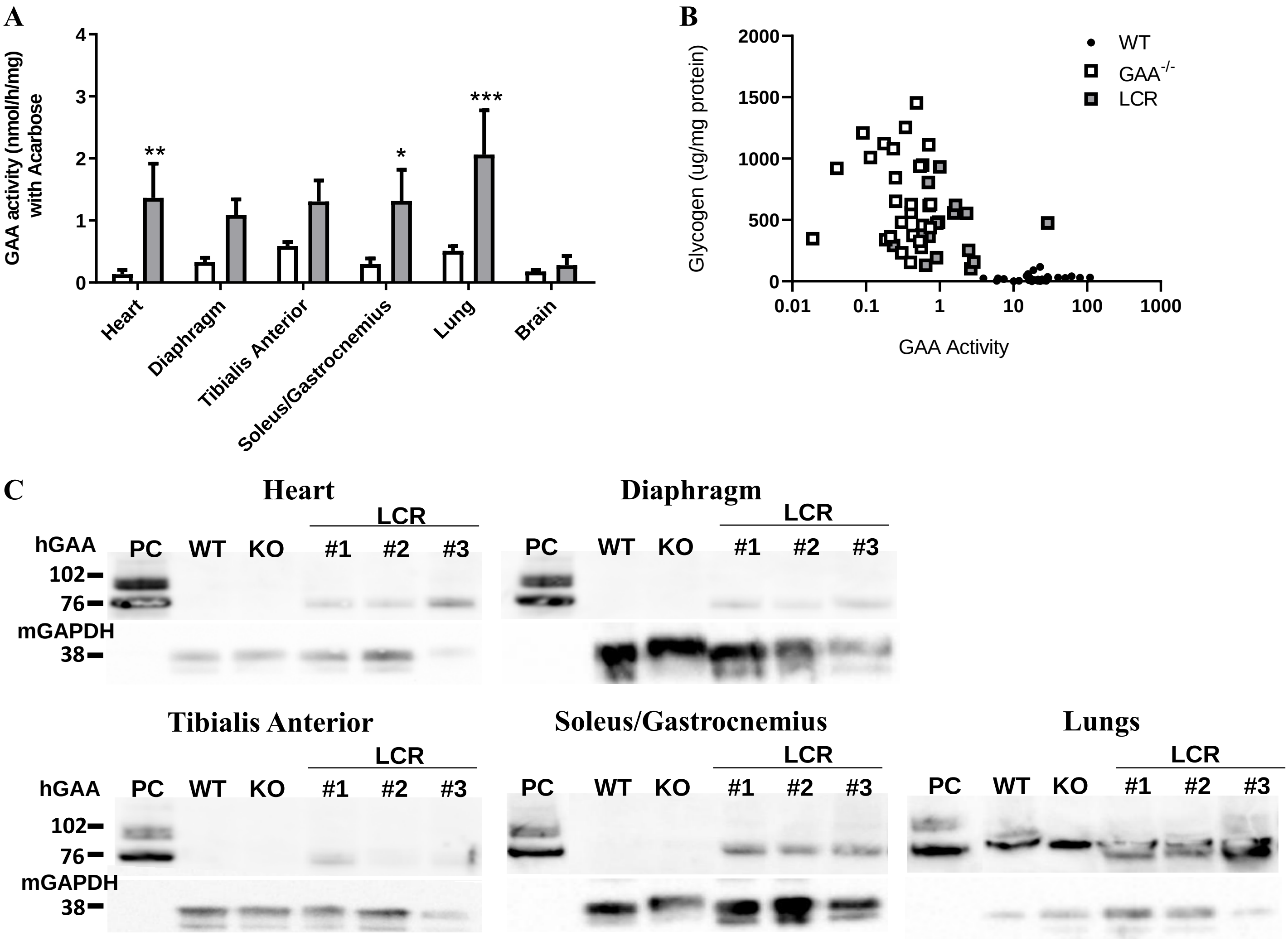

Figure S3. hGAA detected in tissues of treated GAA-/- mice. A) GAA activity measured in tissue homogenates of GAA-/- control mice (white bar) or their littermates treated with LV.LCR-EFS.GAA HSC gene therapy (LCR; grey bar). Data are shown as means ± SEM of 2 independent experiments with n=3-6 mice per group. **B)** Scatter plot displaying the relation between glycogen content and GAA activity in tissues of wild type (black dot), GAA -/- (white square) and LCR treated mice. **C)** Western blot of hGAA in heart, diaphragm, tibialis anterior, soleus/gastrocnemius or lung homogenates of wild type, GAA-/- and LCR treated mice. mGAPDH blot was used as loading control. PC: lysate of LCR transduced 293T cells used as positive control for hGAA.

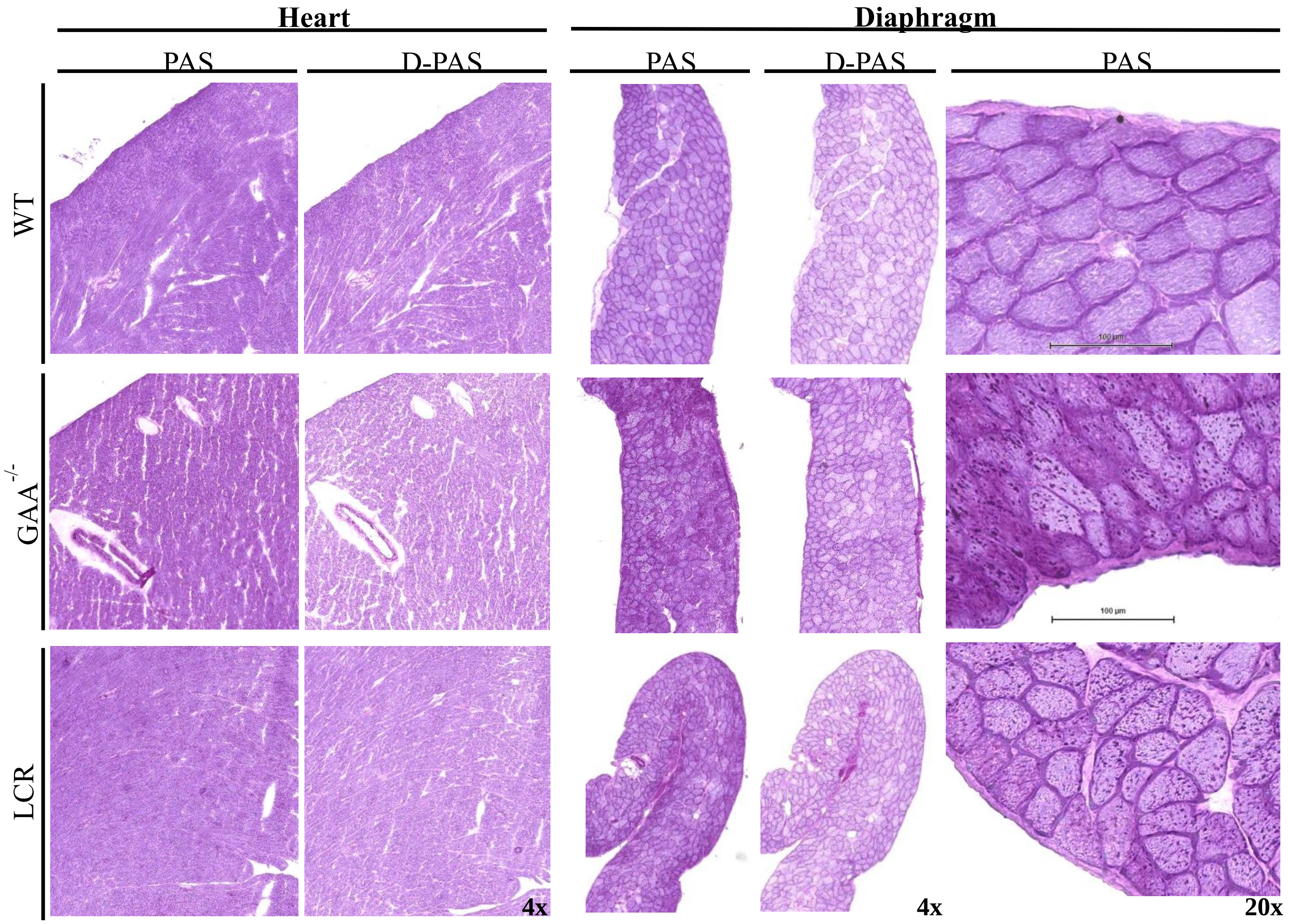

Figure S4. Histology of heart and diaphragm: glycogen staining. Representative images of PAS/ D-PAS staining of heart and diaphragm of wild type, GAA-/- and LV.LCR-EFS.GAA (LCR) treated mice.

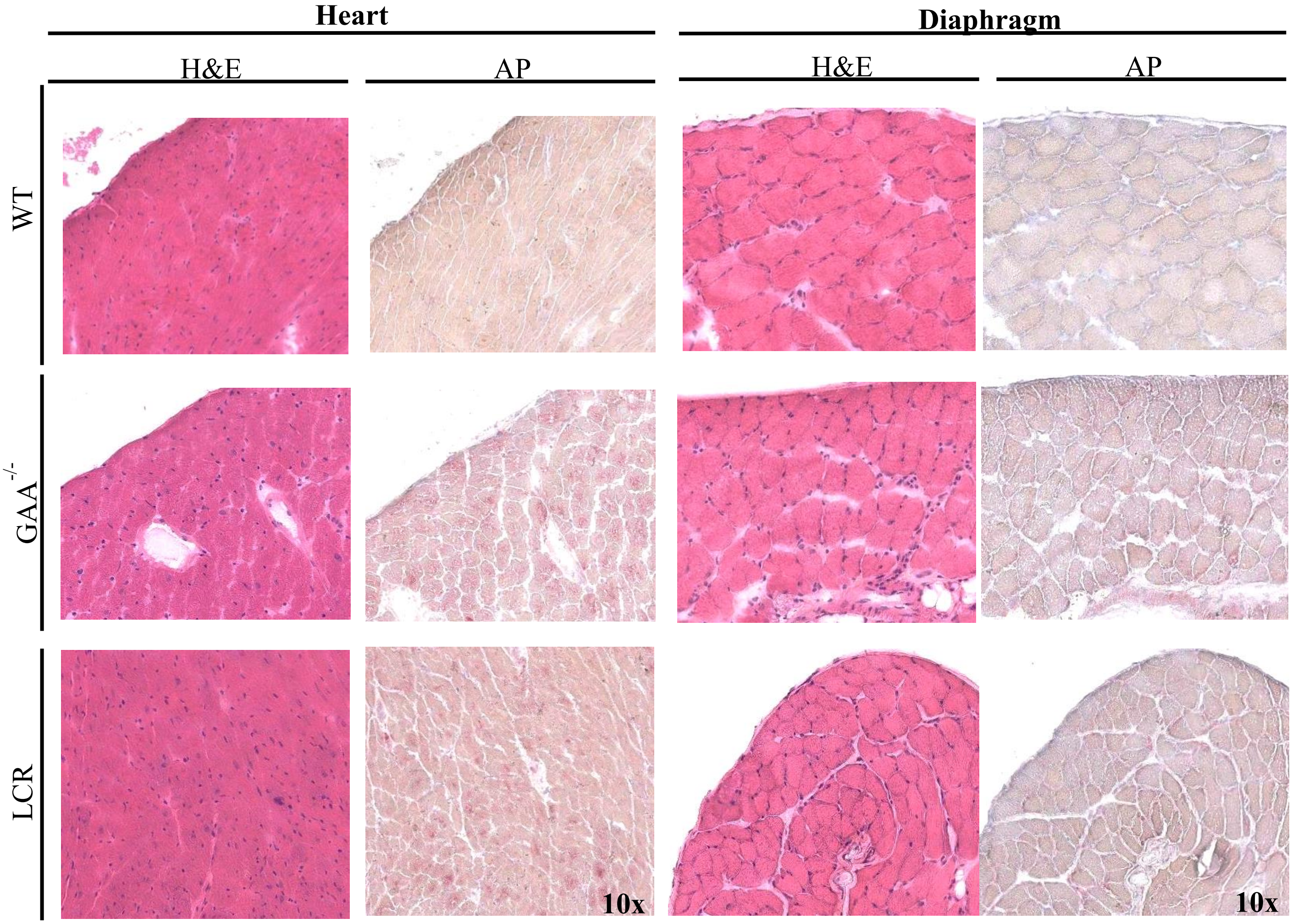

Figure S5. Histology of heart and diaphragm: assessment of pathology. Representative images of H&E and Acid Phosphatase (AP) staining of heart and diaphragm of wild type, GAA-/- and LV.LCR-EFS.GAA (LCR) treated mice.

Table S1. Details on the experimental conditions for LV.LCR-EFS.GAA modified animals

				Reconstitution		PBMC 3mo		PBMC 6mo		Bone marrow 6mo			
ID	Genotype	Gender	Conditioning	Cell type	Cell Number	VCN	Y Chr	VCN	Y Chr	VCN	Y Chr	Bu Toxicity[a]	Tested
TP1_1	GAA$^{-/-}$	F	25mg/kg/day	Lin-	0.5x10^{6}	-	-	-	-	-	-	Yes	No
**TP1_2**	**GAA$^{-/-}$**	**F**	**25mg/kg/day**	**Lin-**	**0.5x10^{6}**	**0.87**	**100%**	**1.2**	**100%**	**0.42**	**52%**	**No**	**Yes**
**TP1_3**	**GAA$^{-/-}$**	**F**	**25mg/kg/day**	**Lin-**	**0.5x10^{6}**	**1.44**	**100%**			**1.22**	**53%**	**No**	**Yes**
TP1_4	GAA$^{-/-}$	F	25mg/kg/day	Lin-	0.5x10^{6}	-	-	-	-	-	-	Yes	No
TP1_5	GAA$^{-/-}$	F	25mg/kg/day	Lin-	0.5x10^{6}	-	-	-	-	-	-	Yes	No
TP2_1	GAA$^{-/-}$	F	25mg/kg/day	Lin-	0.5x10^{6}	0.03	0	0.02	0	0.001	0	No	No
**TP2_2**	**GAA$^{-/-}$**	**F**	**25mg/kg/day**	**Lin-**	**0.5x10^{6}**	**1.05**	**78%**	**1.11**	**82%**	**1.43**	**65%**	**No**	**Yes**
TP2_3	GAA$^{-/-}$	F	25mg/kg/day	Lin-	0.5x10^{6}	-	-	-	-	-	-	Yes	No
TP2_4	GAA$^{-/-}$	F	25mg/kg/day	Lin-	0.5x10^{6}	-	-	-	-	-	-	Yes	No
TP2_5	GAA$^{-/-}$	F	25mg/kg/day	Lin-	0.5x10^{6}	-	-	-	-	-	-	Yes	No
TP2_6	GAA$^{-/-}$	F	25mg/kg/day	Lin-	0.5x10^{6}	-	-	-	-	-	-	Yes	No
TP2_7	GAA$^{-/-}$	F	25mg/kg/day	Lin-	0.5x10^{6}	-	-	-	-	-	-	Yes	No
TP2_8	GAA$^{-/-}$	F	25mg/kg/day	Lin-	0.5x10^{6}	-	-	-	-	-	-	Yes	No
TP2_9	GAA$^{-/-}$	F	25mg/kg/day	Lin-	0.5x10^{6}	-	-	-	-	-	-	Yes	No
TP2_10	GAA$^{-/-}$	F	25mg/kg/day	Lin-	0.5x10^{6}	-	-	-	-	-	-	Yes	No
**TP4_1**	**GAA$^{-/-}$**	**F**	**6 Gy + 4 Gy**	**LSK+**	**1.2x10^{5}**	**0.55**	**55%**	**0.37**		**-**	**-**	**No**	**Yes**
**TP4_2**	**GAA$^{-/-}$**	**F**	**6 Gy + 4 Gy**	**LSK+**	**1.2x10^{5}**	**0.27**	**36%**	**0.34**		**-**	**-**	**No**	**Yes**
**TP4_3**	**GAA$^{-/-}$**	**F**	**6 Gy + 4 Gy**	**LSK+**	**1.2x10^{5}**	**0.50**	**43%**	**-**	**-**	**-**	**-**	**No**	**Yes**
**TP4_4**	**GAA$^{-/-}$**	**F**	**6 Gy + 4 Gy**	**LSK+**	**1.2x10^{5}**	**0.22**	**49%**	**0.3**		**-**	**-**	**No**	**Yes**
**TP4_5**	**GAA$^{-/-}$**	**F**	**6 Gy + 4 Gy**	**LSK+**	**1.2x10^{5}**	**0.30**	**49%**	**0.2**		**-**	**-**	**No**	**Yes**

a) Most of the animals receiving 25mg/kg/day Busilvex developed bloated abdomen linked to gastro-intestinal inflammation. Busilvex, used in clinical trials for HSPC transplants and preclinical study using C57BL/6 mice, revealed toxicity when used to transplant wild type mice in our genetic background, indicating that mice of this strain background do not tolerate the formulation of Busilvex.

Table S2. **Glycogen storage measured post-mortem in different tissues. Percentage of reduction of glycogen storage from knock-out control group in LV.LCR-EFS.GAA (LCR) transplanted group.**

**Glycogen**	**WT**			**GAA$^{-/-}$**			**LCR**			**Glycogen Reduction (%)**
	Mean	SEM	N	Mean	SEM	N	Mean	SEM	N	
Heart	20.5	14.17	6	809.86	156.03	5	270.39	50.78	3	66.6
Diaphragm	46.13	16.1	6	976.90	133.44	5	765.28	110.33	3	21.7
Tibialis Anterior	24.38	7.09	5	854.78	185.57	5	526.37	46.74	3	38.4
Soleus/Gastrocnemius	24.02	3.09	6	654.95	107.28	5	548.79	41.04	3	16.2
Lung	14.27	3.72	6	285.13	47.35	5	130.48	16.26	3	54.4
Brain	2.81	0.62	3	351.15	11.55	2	334.76	42.39	2	-
